# Enhancing photoelectrochemical catalytic performance of zeolite-Co_3_O_4_ composites through optimized ball milling duration

**DOI:** 10.1038/s41598-026-44358-y

**Published:** 2026-05-09

**Authors:** Ghadah M. Al-Senani, Mohamed Shaban, Salhah D. Al-Qahtani, Khaled Abdelkarem, Rana Saad

**Affiliations:** 1 Department of Chemistry, College of Science, Princess Nourah bint Abdulrahman University, P.O. Box 84428, Riyadh, 11671 Saudi Arabia; 2 Department of Physics, Faculty of Science, Islamic University of Madinah, P.O. Box: 170, Madinah, 42351 Saudi Arabia; 3 Department of Physics, Chonnam National University, Gwangju, 61186 Republic of Korea; 4 Nanophotonics and Applications (NPA) Lab, Department of Physics, Faculty of Science, Beni-Suef University, Beni-Suef, 62514 Egypt

**Keywords:** Chemistry, Energy science and technology, Materials science, Nanoscience and technology

## Abstract

This study investigates the impact of ball milling duration on the structural, optical, and photoelectrochemical properties of zeolite–Co_3_O_4_ composites. Catalysts were synthesized using varying ball milling times and characterized using FESEM, FTIR, UV–Vis spectroscopy, EDX, and XRD, revealing that increased ball milling enhances Co_3_O_4_ dispersion within the zeolite matrix, leading to improved light absorption, reduced bandgap energy, and enhanced photocatalytic activity. Furthermore, the photoelectrochemical response of the Cat 4 photocatalyst was investigated under monochromatic light irradiation (390–636 nm) in a 0.5 M Na_2_SO_3_⋅5H_2_O electrolyte at room temperature. The photocurrent density (J_ph_) varied with wavelength, peaking at − 1.62 mA/g at 636 nm and dipping to − 1.55 mA/g at 470 nm, demonstrating significant absorption and effective use of visible light. The incident photon–to–current conversion efficiency (IPCE%) was evaluated, revealing a peak value of 4.98% at 390 nm and a minimum of 3.16% at 636 nm. The applied bias photon–to–current efficiency (ABPE%) analysis demonstrated a maximum efficiency of 0.098% at 636 nm with an applied potential of 0.56 V vs. RHE. Photoelectrochemical measurements demonstrate that the catalyst with the longest ball milling duration exhibits the highest photocurrent density and hydrogen evolution rate, confirming the catalyst’s effective charge carrier separation and transport, contributing to enhanced solar–to–hydrogen energy conversion, and indicating its superior performance in PEC water splitting.

**Supplementary Information:**

The online version contains supplementary material available at 10.1038/s41598-026-44358-y.

## Introduction

The combustion of fossil fuels is a primary source of atmospheric CO_x_ emissions (CO_2_ and CO), significantly contributing to global warming and posing severe environmental and health risks. As a sustainable and clean energy carrier, hydrogen has emerged as a viable alternative to fossil fuels, necessitating the development of efficient and cost-effective hydrogen production technologies. Among these, photoelectrochemical (PEC) water splitting, which employs metal oxide-based photocatalysts, is a promising strategy for hydrogen generation^1^. Incident photons excite the photocatalyst in this process, generating electron-hole pairs that drive water splitting into hydrogen and oxygen. The increasing global demand for renewable energy has intensified research efforts to enhance the efficiency of PEC materials. Zeolite-based composites have garnered significant interest due to their structural adaptability, enabling the incorporation of active components such as Co_3_O_4_ to improve light absorption and charge transport. Consequently, the development of cost-effective and high-performance PEC catalysts using earth-abundant materials remains a crucial objective in advancing sustainable hydrogen production^2^.

Zeolites are crystalline microporous aluminosilicate materials composed of interconnected SiO_2_ and Al_2_O_3_ tetrahedra, forming a highly ordered framework with ion-exchangeable cations and water molecules within their porous network^3,4^. Their exceptional physicochemical properties, including a tunable semiconducting nature, high ion exchange capacity, large specific surface area, remarkable chemical and thermal stability, adjustable hydrophobicity/hydrophilicity, and non-toxicity, have made them versatile materials for various applications^5^. Zeolites have been extensively utilized as stable supports for metal nanoparticles due to their ability to facilitate nanoparticle dispersion and prevent aggregation, thereby enhancing catalytic efficiency. Furthermore, their strong adsorption capabilities have positioned them as promising candidates for pollutant removal, gas separation, and sensor applications^6^. Recent studies have also explored the impact of different exchangeable cations on the adsorption, ion exchange, and charge transport properties of zeolites, further broadening their potential in energy storage, environmental remediation, and photoelectrochemical processes. These characteristics make zeolites highly attractive for advanced functional materials and catalytic applications^7,8^.

The use of zeolite-based materials in photocatalytic applications, especially PEC water splitting, has garnered significant attention, with zeolites acting as stable support for active components and offering unique structural advantages^9^. Integrating Co_3_O_4_ into zeolite frameworks is a promising strategy to enhance PEC water-splitting efficiency. Zeolites, known for their high surface area and ion-exchange capabilities, serve as effective supports for dispersing Co_3_O_4_ nanoparticles, thereby improving light absorption and charge separation^10^. Research efforts have focused on modifying zeolite structures and optimizing synthesis methods to maximize the photocatalytic performance of these composite materials. Recent studies have demonstrated that such composites exhibit superior PEC performance compared to individual components^11^.

Although Co_3_O_4_-based materials have been extensively studied for photocatalysis and PEC water splitting, the literature shows a clear gap regarding the role of mechanical activation, especially ball milling, in tailoring Co_3_O_4_–matrix composites. Most Co_3_O_4_ photocatalysts are synthesized using hydrothermal, sol–gel, chemical precipitation, or electrodeposition routes^12,13^, which control morphology but do not deliberately induce defect states, oxygen vacancies, lattice restructuring, or interfacial bonding modifications. Existing reports on mechanical activation focus mainly on crystallite size reduction or powder homogenization^14,15^, with almost no studies examining how milling-induced defect chemistry, framework rearrangement, or interfacial Co–O–Si/Al interactions influence PEC charge transport. Furthermore, no systematic work has correlated different ball-milling durations with changes in bandgap, pore architecture, and PEC hydrogen evolution efficiency in zeolite–Co_3_O_4_ composites. This gap highlights the novelty of the present study, demonstrating that controlled mechanical activation is a powerful strategy to tune structural and electronic properties in Co_3_O_4_-based composites for significantly enhanced PEC performance^10^.

This study investigates the photocatalytic performance of cobalt oxide-zeolite (Co_3_O_4_-Zeolite) composite for water splitting under simulated sunlight. The Co_3_O_4_-Zeolite photocatalyst was synthesized and subjected to comprehensive characterization to analyze its structural, optical, and electronic properties. Electrochemical measurements were conducted in a three-electrode setup consisting of a graphite working electrode, a platinum counter electrode, and an Ag/AgCl reference electrode to evaluate its hydrogen evolution activity. The photocatalyst was dispersed in a sodium sulfite heptahydrate electrolyte to facilitate redox reactions. To assess its wavelength-dependent photoresponse, experiments were performed using bandpass filters covering 390–636 nm, enabling an in-depth evaluation of key performance parameters such as photocurrent density, incident photon-to-current conversion efficiency (IPCE), and applied bias photon-to-current efficiency (ABPE). The results demonstrate that the Co₃O₄-Zeolite composite exhibits strong activity in the visible light region, showing enhanced photocurrent responses at specific wavelengths. This research contributes to the development of high-performance photocatalytic systems by leveraging the synergistic interaction between cobalt oxide and the zeolite framework, which enhances light absorption, charge separation, and overall catalytic efficiency. Understanding the correlation between the composite’s structural features and its PEC performance is essential for designing next-generation materials for efficient solar-to-hydrogen conversion. In conclusion, this study highlights the potential of Co_3_O_4_-Zeolite as a photocatalyst for sustainable hydrogen production and provides valuable insights for the advancement of PEC water-splitting technology.

## Experimental work

### Catalysts preparation

The simplicity of the preparation method for PEC catalysts is crucial for industrial applications, including hydrogen generation. A natural clinoptilolite zeolite sample was sourced from Al Nasr Company for Mining, Egypt. The sample underwent ball milling for 2, 4, 6, and 8 h at 700 rpm, producing fine powders with varying degrees of fineness. Five separate 5-gram samples of the natural and milled zeolite from each milling duration were then mixed with 50 mL of a 0.5 M aqueous solution of Cobalt (II) acetate tetrahydrate (Co(C₂H₃O₂)₂·4 H₂O, 99.5%, PIOCHEM, Egypt). This mixing was performed using a hot plate magnetic stirrer set at 1000 rpm for 30 min.

To ensure uniform cobalt ion loading, the suspensions were ultrasonicated for 30 min, followed by filtration, washing with distilled water, and drying at room temperature. Following the ultrasonication process, the Co-loaded zeolite samples were filtered, washed with distilled water, and dried at room temperature. To convert the loaded cobalt ions into cobalt oxide, the samples were subjected to thermal treatment at 500 °C for 3 h. After ball milling and thermal treatment, partial structural transformations occurred within the zeolite framework. As confirmed by XRD, additional zeolite phases such as Zeolite A, Zeolite Beta, and Zeolite Nu-3 appeared alongside the parent clinoptilolite. These transformations are attributed to framework rearrangement and amorphization–recrystallization processes during high-energy milling and calcination. This procedure resulted in the formation of cobalt oxide-loaded zeolite composites, which were subsequently characterized for further analysis. The samples produced in this study are designated as follows: Cat 1 (natural zeolite/Co_3_O_4_), Cat 2 (zeolite/Co_3_O_4_ ball-milled for 2 h), Cat 3 (zeolite/Co_3_O_4_ ball-milled for 4 h), Cat 4 (zeolite/Co_3_O_4_ ball-milled for 6 h), and Cat 5 (zeolite/Co_3_O_4_ ball-milled for 8 h).

### Characterization of prepared samples

A comprehensive suite of characterization techniques was employed to investigate the physicochemical properties of the synthesized samples. Nanomorphology was examined using Field Emission-Scanning Electron Microscopy (FE-SEM, Sigma 500 VP, Zeiss, Oberkochen, Germany). Fourier Transform Infrared (FTIR) spectroscopy (FTIR–8400 S Shimadzu, Milton Keynes, UK) with a KBr matrix identified functional groups within the 4000 to 400 cm^–1^ range. Elemental composition was determined by energy–dispersive X-ray (EDX) Spectroscopy (AMETEK, Inc., Berwyn, PA, USA) coupled with the SEM detector. Optical properties were probed using UV/Vis spectroscopy (Lambda 950 spectrophotometer, Perkin Elmer). Finally, phase purity and crystallinity were assessed by X–ray diffraction (XRD, Philips X’Pert ProMRD, Malvern, UK) with Cu Kα radiation, scanning from 10° to 50° 2θ operated at 40 kV and 20 mA. Additionally, the specific surface area and pore size distribution of the samples were measured by N₂ adsorption–desorption at 77 K using a Micromeritics ASAP 2020 analyzer. Prior to analysis, all samples were degassed at 200 °C under vacuum for 12 h. BET surface area was calculated from adsorption data in the relative pressure range 0.05–0.3, while the BJH method was applied to the desorption branch of the isotherms. For further pore size analysis, the DFT model was also employed.

### Photoelectrochemical measurement setup

The hydrogen evolution performance was assessed under simulated solar irradiation (AM 1.5G, 100 mW/cm^2^) using a 400 W mercury–xenon light source (Newport, MODEL: 66926–500HX–R07, Newport, UK). Electrochemical measurements were conducted with an OrigaFlex electrochemical station (OGFEIS coupled with an OGF500 Pack, France). A three-electrode configuration was employed, consisting of a graphite working electrode, a platinum counter electrode, and an Ag/AgCl reference electrode. For the experiment, 0.05 g of the photocatalyst was dispersed in 50 mL of a 0.3 M sodium sulfite heptahydrate (Na_2_SO_3_·7H_2_O) solution, which served as the supporting electrolyte.

## Results and discussion

### Surface area, pore volume, and milling time optimization

Optimizing milling time is essential for tailoring zeolite characteristics for specific industrial applications. Table S1 (Supplementary data) presents the BET surface area, BJH pore volume, DFT method surface area, total pore volume at relative pressure 0.99540, and average pore radius for five catalysts. Catalyst 1 has a BET surface area of 72.21 m²/g, a total pore volume of 0.13881 cc/g, and an average pore radius of 3.8447 nm. Catalyst 2, with the highest BET surface area of 108.751 m²/g, has a total pore volume of 0.18991 cc/g and an average pore radius of 3.4927 nm. Catalyst 3 shows a BET surface area of 131.665 m²/g, a total pore volume of 0.14958 cc/g, and an average pore radius of 2.2722 nm. Catalyst 4 features a BET surface area of 47.0813 m²/g, the highest total pore volume of 0.23884 cc/g, and the largest average pore radius of 10.146 nm. Catalyst 5 displays a BET surface area of 54.0373 m²/g, a total pore volume of 0.16242 cc/g, and an average pore radius of 6.0115 nm. This study focused on the characterization and PEC hydrogen production using Cat 1, Cat 2, and Cat 4 due to their distinct properties. Cat 1 was selected for its moderate surface area and pore volume, providing a balanced comparison. Cat 2 was chosen for its highest surface area, crucial for catalytic activity and hydrogen production efficiency. Beyond simply increasing the surface area, the inert zeolite framework provides several synergistic benefits that significantly enhance charge separation in the Co_3_O_4_–Zeolite composite. The aluminosilicate structure of zeolite contains negatively charged AlO⁻ tetrahedra balanced by extra-framework cations (Na^+^, K^+^, Ca^2+^), which create intrinsic electrostatic fields that facilitate the spatial separation of photogenerated electrons and holes. In addition, the zeolite’s microporous channels act as electron-transport corridors, enabling directional movement of carriers while physically restricting the recombination pathways typically present on bulk Co_3_O_4_ surfaces. The strong Co–O–Si/Al interfacial bonding formed during milling further creates heterojunction-like interfaces, where band alignment between Co_3_O_4_ and the insulating zeolite induces a charge-transfer barrier that helps trap holes at Co_3_O_4_ active sites, while migrating electrons are discouraged from recombining. Finally, the dielectric nature of zeolite suppresses rapid electron-hole recombination by limiting direct electron mobility across the composite. These synergistic interactions collectively enhance charge separation efficiency, thereby contributing to the superior PEC performance observed in Cat 4.

Cat 4 was highlighted for its unique combination of the highest pore volume and largest pore radius, significantly impacting its performance in PEC hydrogen production. Cat 4 demonstrated the highest PEC performance due to its large pore radius and high total pore volume, which enhanced the diffusion of reactants and products, thereby improving the overall efficiency of the photoelectrochemical process, as shown in Figure S1 (Supplementary data). This figure illustrates the current density-applied potential (J-V) curves for PEC hydrogen production for all samples, with the inset showing the current density values at −0.2714 V vs. RHE. The current densities at this potential for the five catalysts indicate varying levels of catalytic activity, with Cat 4 exhibiting the highest current density (−2.0224 A/cm²) and Cat 5 the lowest (−0.73507 A/cm²). Cat 1, Cat 2, and Cat 4 were selected for detailed characterization due to their distinct performance profiles: Cat 1 offers moderate activity with potential advantages in stability or cost-effectiveness; Cat 2 balances higher activity with other beneficial properties; and Cat 4, with the highest activity, is ideal for applications requiring robust catalytic performance. This focused analysis aims to optimize their use by understanding their structural, electrochemical, and durability characteristics.

### Optical, morphological, compositional, and structural characteristics

#### UV–visible and FTIR Spectroscopy

**Fig. 1:**
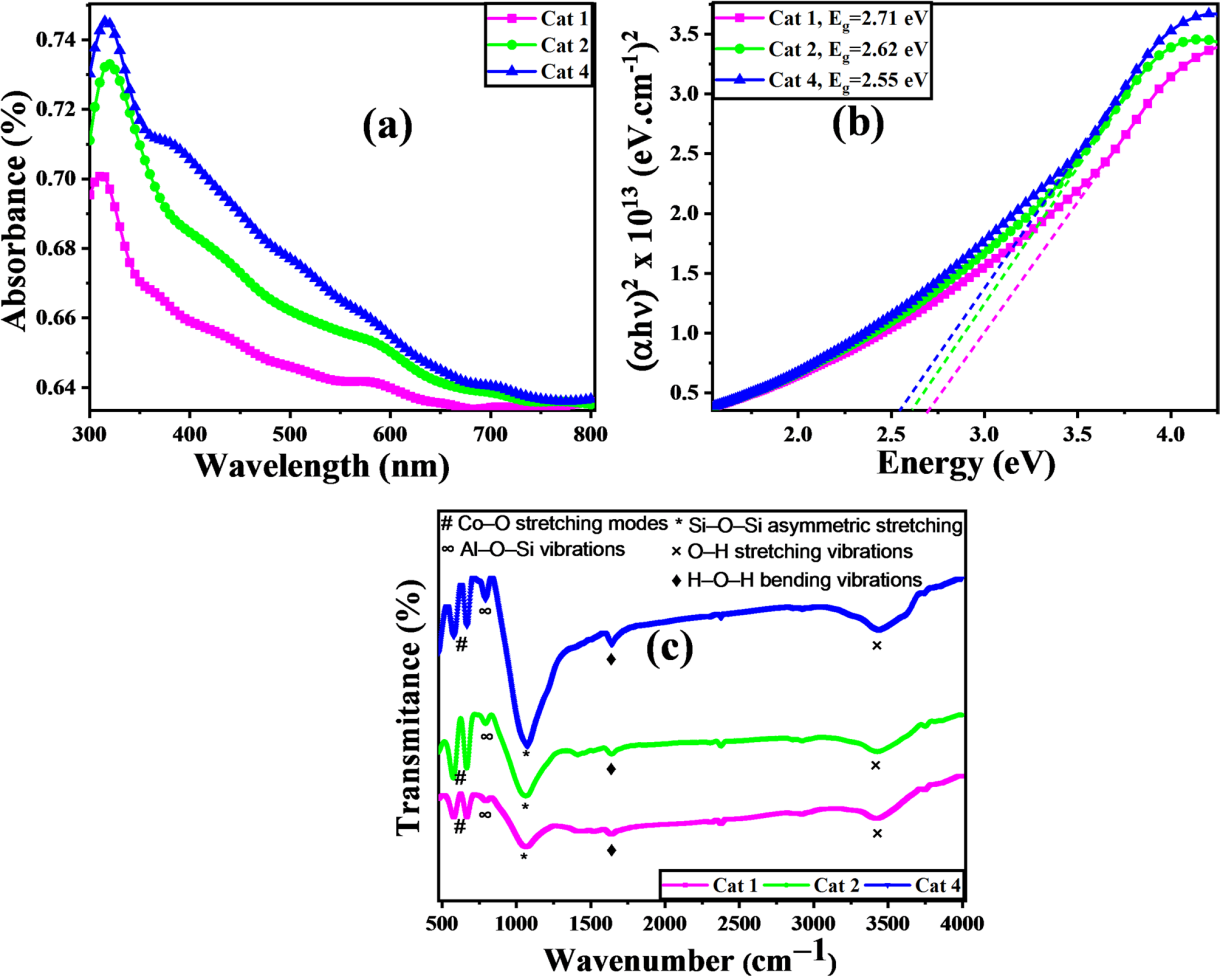
(**a**) Absorbance spectra, (**b**) Band gap, and (**c**) FTIR of Cat 1, Cat 2, and Cat 4 catalyst samples, respectively.

The absorbance spectra of three different catalyst samples (Cat 1, Cat 2, and Cat 4) were investigated using UV–visible spectroscopy to determine their absorbance profiles across the wavelength range of 300 to 800 nm (Fig. 1 (a)). The resulting spectra revealed distinct variations in absorbance intensity and spectral features among the catalysts, suggesting differences in their electronic transitions and light absorption capabilities.

Cat 4 (blue) exhibited the highest absorbance across the measured range, followed by Cat 2 (green), while Cat 1 (violet) demonstrated the lowest absorbance. This indicates a greater capacity for light harvesting in Cat 4, leading to enhanced performance in applications requiring efficient light absorption, such as photocatalysis and optoelectronic devices^9,16^. A strong absorption peak observed around 350 nm for all catalysts suggests significant light absorption in the UV region, characteristic of Co_3_O_4_^17^. These findings suggest that optimizing ball milling duration is a crucial strategy for tailoring the optical properties and enhancing the performance of zeolite–Co_3_O_4_ composites in applications requiring efficient light absorption, such as photocatalysis or optoelectronic devices^18^.

The Tauc plot shown in Fig. 1(b) illustrates the optical bandgap energy () of three different catalyst samples (Cat 1, Cat 2, and Cat 4), determined using UV–Vis spectroscopy and the Tauc equation for direct bandgap semiconductors through extrapolating the linear portion of the (hν)^2^ vs. ℎ curve to the energy axis (hν). Where α is the absorption coefficient and hν is the photon energy. This reveals that Cat 1 exhibits the highest bandgap energy (E_g_ = 2.71 eV), followed by Cat 2 (E_g_ = 2.62 eV), while Cat 4 has the lowest bandgap (E_g_ = 2.55 eV). The decrease in bandgap energy from Cat 1 to Cat 4 is due to enhanced defect states, which facilitate improved charge carrier mobility^19,20^. These variations in bandgap energy imply that Cat 4 has superior light absorption properties in the visible range, making it more suitable for photocatalytic and optoelectronic applications^21,22^.

The FTIR spectra in Fig. 1(c) illustrate the impact of increasing ball milling duration on the structural and chemical properties of cobalt–zeolite catalysts prepared with varying ball milling times (Cat 1 – natural, Cat 2–2 h, and Cat 4–6 h). The spectra also exhibit several characteristic vibrational features that reflect both the intrinsic aluminosilicate framework and the incorporation of cobalt oxide species. A broad and intense band in the range of 3400–3600 cm^− 1^ is assigned to the O–H stretching vibrations of surface hydroxyl groups and physiosorbed water molecules within the microporous channels of the zeolite^23–25^. The broadening and slight red-shift of this band with increasing milling duration (Cat 1 → Cat 4) indicate an increase in surface defect density and structural disorder, which enhances the number of hydroxylated sites capable of interacting with ambient moisture. Additionally, the band observed near 1620–1650 cm^− 1^ corresponds to the H–O–H bending vibrations of molecularly adsorbed water confined within the zeolite cavities, further confirming the hydrophilic nature of the aluminosilicate framework.

The strong absorption feature centered between 1000 and 1200 cm^− 1^ is attributed to the asymmetric stretching mode of the Si–O–Si^26,27^, representing the primary structural backbone of the zeolite lattice. Subtle variations in peak position and intensity across the samples reflect modifications in bond angles and local symmetry caused by high-energy ball milling and subsequent cobalt loading. The presence of the Si–O–Al and Al–O–Si vibrations within the 800 cm⁻¹ validates the framework substitution of Al in tetrahedral coordination. These bands exhibit measurable changes in broadness and relative intensity for Cat 4, suggesting partial amorphization or rearrangement of the tetrahedral units due to prolonged milling, which is consistent with observed XRD structural evolution. Bands located between 500 and 700 cm^− 1^ correspond to the Co–O stretching vibrations originating from surface and lattice modes of cobalt oxide species (primarily spinel-Co_3_O_4_). These include contributions from Co²⁺–O tetrahedral sites (≈ 560–580 cm^− 1^) and Co³⁺–O octahedral sites (≈ 630–690 cm^− 1^)^28,29^. The progressive intensification of these Co–O bands with milling duration demonstrates enhanced dispersion and incorporation of cobalt ions within the zeolitic framework, as well as improved interfacial contact between the metal oxide and the aluminosilicate skeleton. This observation corroborates EDX analysis, which indicates increased cobalt distribution and successful anchoring onto the modified surface.

These changes increase ball milling duration, enhance cobalt–zeolite interaction, leading to improvements in catalytic activity, adsorption capacity, and overall material performance in applications such as gas sensing and photocatalysis^30,31^.

#### Morphologies and chemical composition

**Fig. 2:**
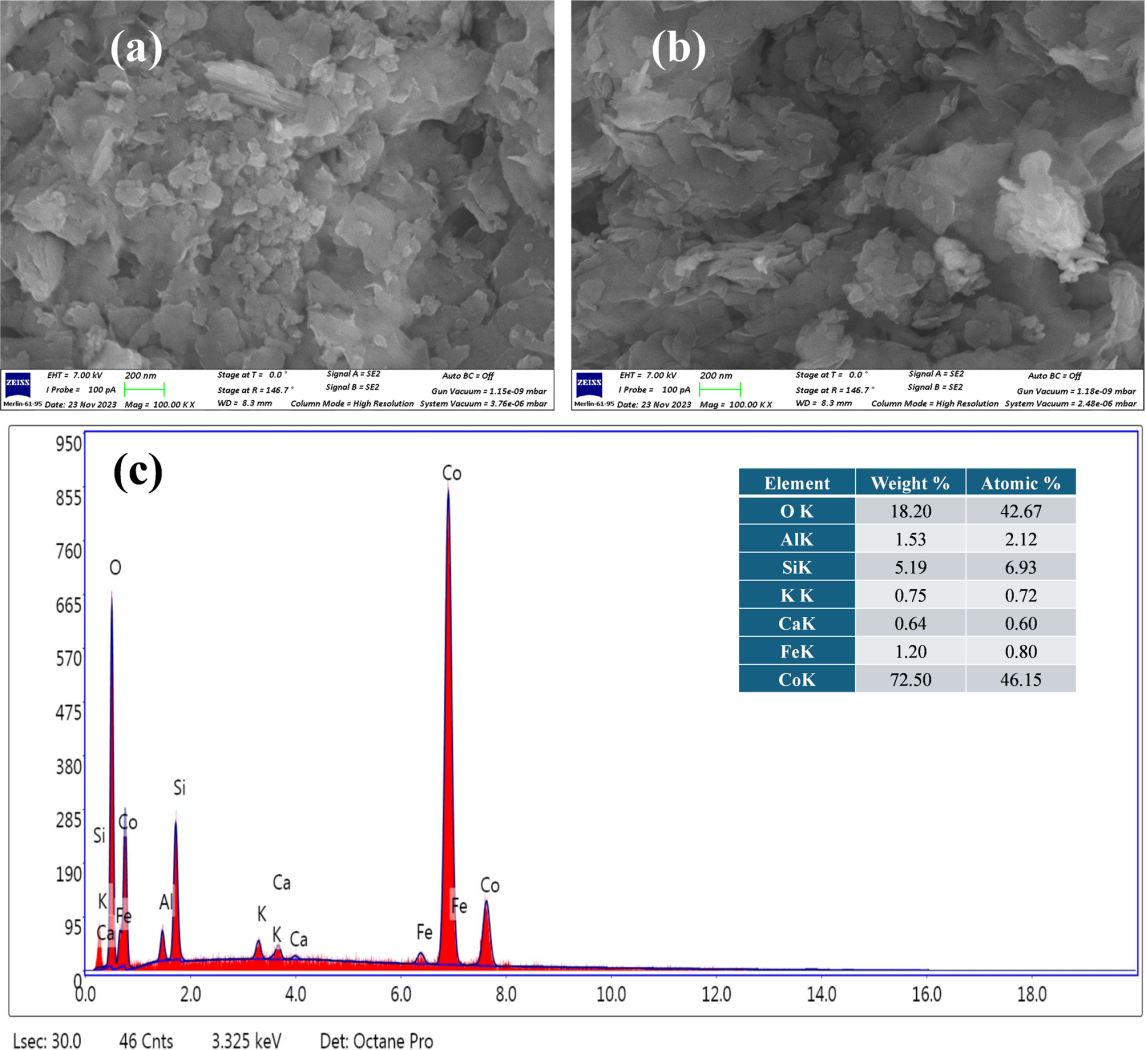
(**a**) and (**b**) SEM images for Cat 2 and Cat 4, respectively, and (**c**) EDX for Cat 4.

SEM analysis revealed the impact of ball milling duration on the morphology of zeolite–Co_3_O_4_ composites (Fig. 2 (a, b)). The SEM image of natural zeolite (Cat 1), Figure S2(a) (supplementary data), showcases its detailed microstructure and characteristic layered formation, which is common for this type of mineral. Cat 2, prepared with 2 h of ball milling, exhibited irregular agglomerates and plate-like structures, indicative of a less uniform dispersion of Co_3_O_4_ within the zeolite matrix. This morphology presents both advantages and disadvantages for photoelectrochemical performance. The high surface area of the agglomerates can enhance light absorption and provide abundant active sites^32,33^ the increased grain boundaries can hinder charge transport^34^.

The extended ball milling duration observed in Cat 4 leads to a more refined and homogenized texture, characterized by smaller agglomerates, well–defined plate-like features, reduced particle sizes, and enhanced dispersion of Co_3_O_4_ within the zeolite matrix^35,36^. This enhanced dispersion increases the surface area, providing more active sites for water oxidation and reduction reactions. Additionally, the more defined plate-like structures in Cat 4 facilitate improved charge transport by minimizing grain boundaries and creating efficient pathways for electron–hole separation and transfer^37^.

Zeolite is divided into heulandite and clinoptilolite, which are isostructural with each other. The EDX spectrum and elemental composition analysis of the natural zeolite (Cat 1) are presented in Figure S2(b) (supplementary data). As seen, this spectrum displays the three main elements (Si, Al, and O). In addition to a small trace from Fe, Cu, K, and Ca are observed. The Si/Al ratio of 3.54 suggests the presence of Heulandite, as this mineral typically exhibits Si/Al ratios ranging from 2.8 to 4^33^. The EDX spectrum and elemental composition analysis of the cobalt–zeolite catalyst (Cat 4) show that cobalt was successfully added to the zeolite framework. The spectrum displays peaks corresponding to various elements, with the table quantifying their weight and atomic percentages. The prominent CoK peak indicates a high cobalt content (72.50 wt%, 46.15 at%), suggesting significant cobalt loading, which is crucial for enhancing its catalytic activity^38,39^. Oxygen (18.20 wt%) is present due to the zeolite structure and cobalt oxides, while silicon (5.19 wt%) and aluminum (1.53 wt%) correspond to the zeolite framework. Minor elements, such as potassium, calcium, and iron, indicate residual impurities that influence the catalytic properties^40^. The high cobalt concentration suggests the catalyst has strong potential for catalytic applications, especially in redox reactions and adsorption–based processes^41,42^.

#### XRD study

**Fig. 3:**
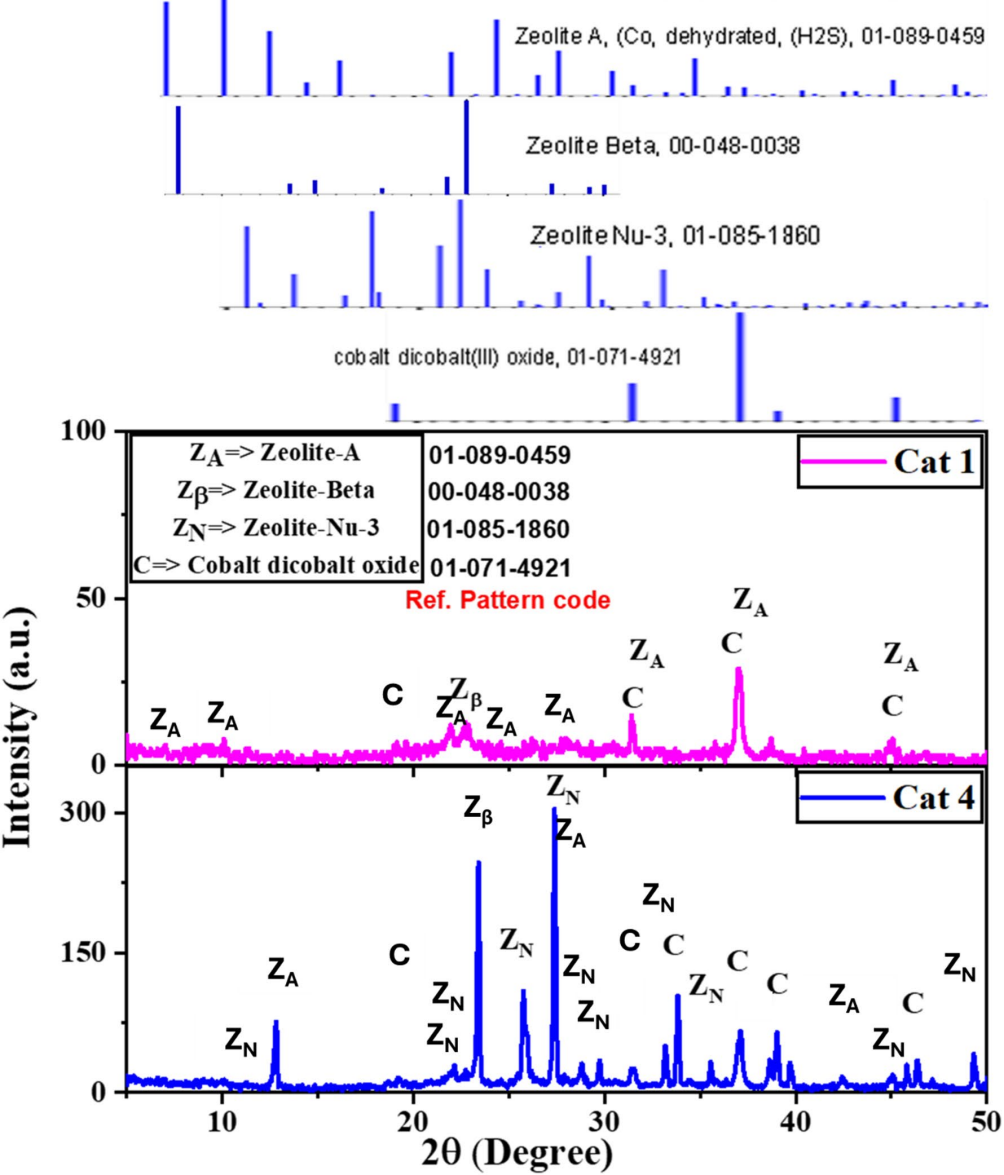
XRD patterns of Cat 1 and Cat 4 compared with standard JCPDS references for Zeolite-A (01–089-0459), Zeolite-Beta (00–048-0038), Zeolite-Nu-3 (01–085-1860), and cobalt oxide (01–071-4921), confirming the crystalline phases present in each catalyst.

The crystalline structures of Cat 1 and Cat 4 were examined using XRD, and the indexed diffraction patterns are presented in Fig. 3. The reference patterns for Zeolite-A (JCPDS 01–089-0459), Zeolite-Beta (JCPDS 00–048-0038), Zeolite-Nu-3 (JCPDS 01–085-1860), and cobalt oxide (JCPDS 01–071-4921) were used to assign the major reflections. The XRD chart of Cat 3 is shown in Figure S3 (Supplementary data). Although the starting material was clinoptilolite, the combined effects of high-energy milling and calcination induced partial framework rearrangement, consistent with previous reports on the structural sensitivity of natural zeolites under mechanical and thermal stress^43–46^. For Cat 1, the diffraction peaks at 2θ ≈ 7.2° (100), 10.2° (110), 12.6° (111), 21.9° (300), 24.3 (311) and 27.6° (321) match the indexed planes of Zeolite-A, while additional reflections at 2θ ≈ 14.8° (100), 21.8° (302), 22.8°(403), and 27.4°(511), correspond to Zeolite-Beta. The relatively broad and low-intensity peaks indicate reduced crystallinity, likely due to partial amorphization and defect formation during milling^3^. Peaks at 2θ ≈ 19.0° (111), 31.3° (220), 37.0° (311), 38.8° (222) and 45.1° (400) confirm the presence of cobalt oxide, consistent with cobalt incorporation into the composite^47,48^ Cat 4 exhibits additional reflections at 2θ ≈ 11.1°(012), 22.2° (122), 23.5°(300), 25.4°(205), 27.4°(220), 28.8°(125), 29.6°(312), 32.7°(042), 35.1°(315), 45.2°(152) and 49.2°(155) indexed to Zeolite-Nu-3, indicating further framework transformation. These changes align with earlier studies showing that natural zeolites can reorganize into new crystalline phases under mechanical activation and heat treatment^49,50^. The increased intensity and narrower width of these peaks reflect an improved degree of crystallinity, and Cat 4 results in a highly ordered framework. The cobalt dicobalt oxide phase detected in Cat 4 indicates the incorporation of cobalt species into the zeolite structure, which impacts catalytic activity, adsorption behavior, and electronic properties^51^. The increased crystallinity in Cat 4 enhanced structural stability, thereby improving its performance in photocatalytic applications^52^.

#### XPS analysis of the Zeo/Co₃O₄ catalyst

**Fig. 4:**
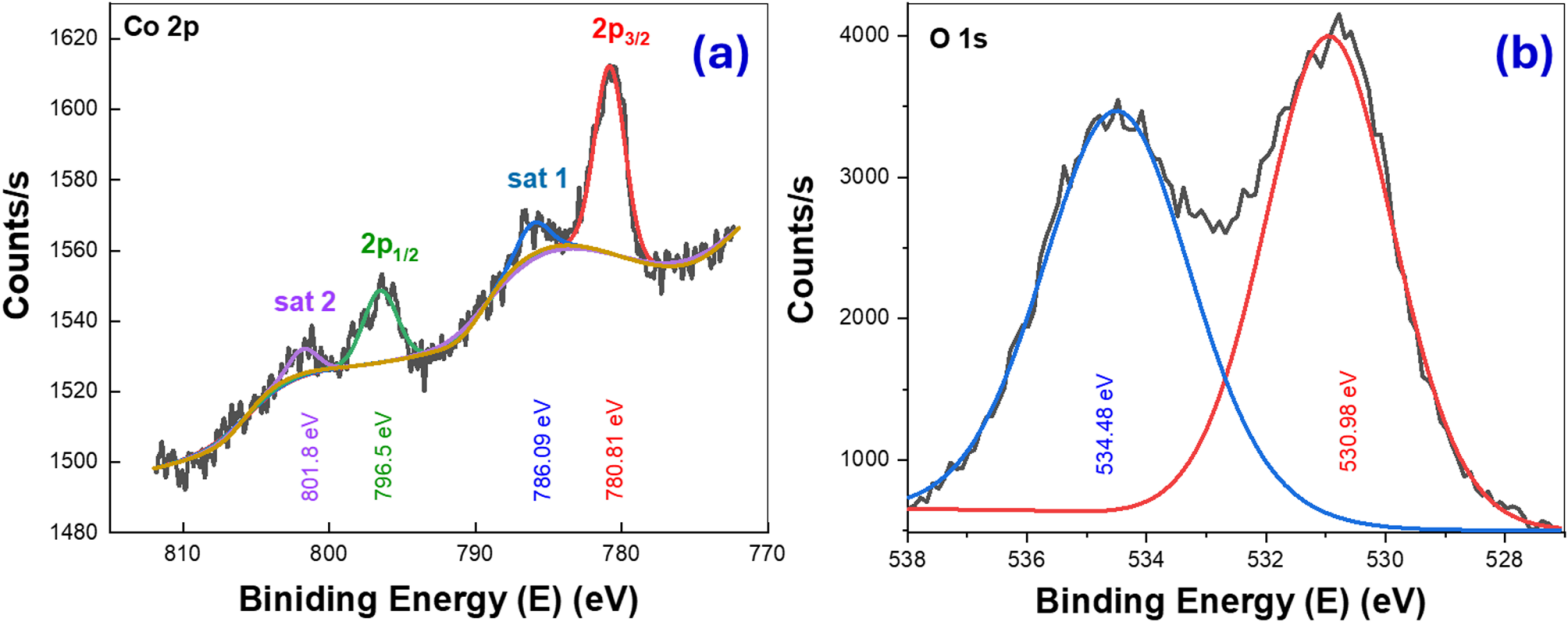
XPS charts for (**a**) Co2p and (**b**) O1s.

X-ray photoelectron spectroscopy was employed to investigate the surface chemistry of the Zeo/Co₃O₄ catalyst, with particular attention to the Co 2p and O 1 s regions, Fig. 4. The Co 2p spectrum, Fig. 4(a), exhibits the characteristic features of spinel-type cobalt oxide, confirming that Co₃O₄ is successfully incorporated onto the zeolite surface. The Co 2p₃/₂ peak appears at ≈ 780.8 eV, accompanied by a shake-up satellite at ≈ 786.1 eV, which is indicative of Co²⁺ species. The corresponding Co 2p₁/₂ signal at ≈ 796.5 eV, together with its satellite at ≈ 801.8 eV, reflects the presence of Co³⁺. The separation between the main peaks and their satellites, along with the overall spectral shape, is consistent with the mixed-valence configuration of Co₃O₄, where Co²⁺ occupies tetrahedral sites and Co³⁺ resides in octahedral coordination. The quality of the spectral fitting suggests that cobalt oxide is uniformly dispersed on the zeolite without contributions from metallic cobalt or secondary cobalt oxide phases.

The O 1 s region, Fig. 4(b), provides further insight into the oxygen environment within the composite. The primary component at ≈ 531.0 eV corresponds to lattice oxygen (O²⁻) associated with both the Co–O bonds of Co₃O₄ and the aluminosilicate framework of the zeolite. A second, higher-binding-energy peak at ≈ 534.5 eV is attributed to surface hydroxyl groups, adsorbed water molecules, and oxygen-related defect species. The presence of these surface oxygen species suggests a defect-rich environment, commonly associated with oxygen vacancies or under-coordinated oxygen sites. Such features are known to facilitate hole trapping and enhance interfacial charge transfer during PEC water oxidation.

Taken together, the Co 2p and O 1 s spectra confirm the coexistence of Co²⁺/Co³⁺ redox couples and the presence of both structural and defect-related oxygen species. This combination is advantageous for PEC hydrogen generation, as mixed-valence cobalt centers and oxygen vacancies promote efficient charge separation and accelerate surface redox kinetics, ultimately contributing to the enhanced performance of the Zeo/Co₃O₄ catalyst.

### Photoelectrochemical performance of zeolite–based catalysts

#### Photoelectrochemical current density vs. applied potential

**Fig. 5:**
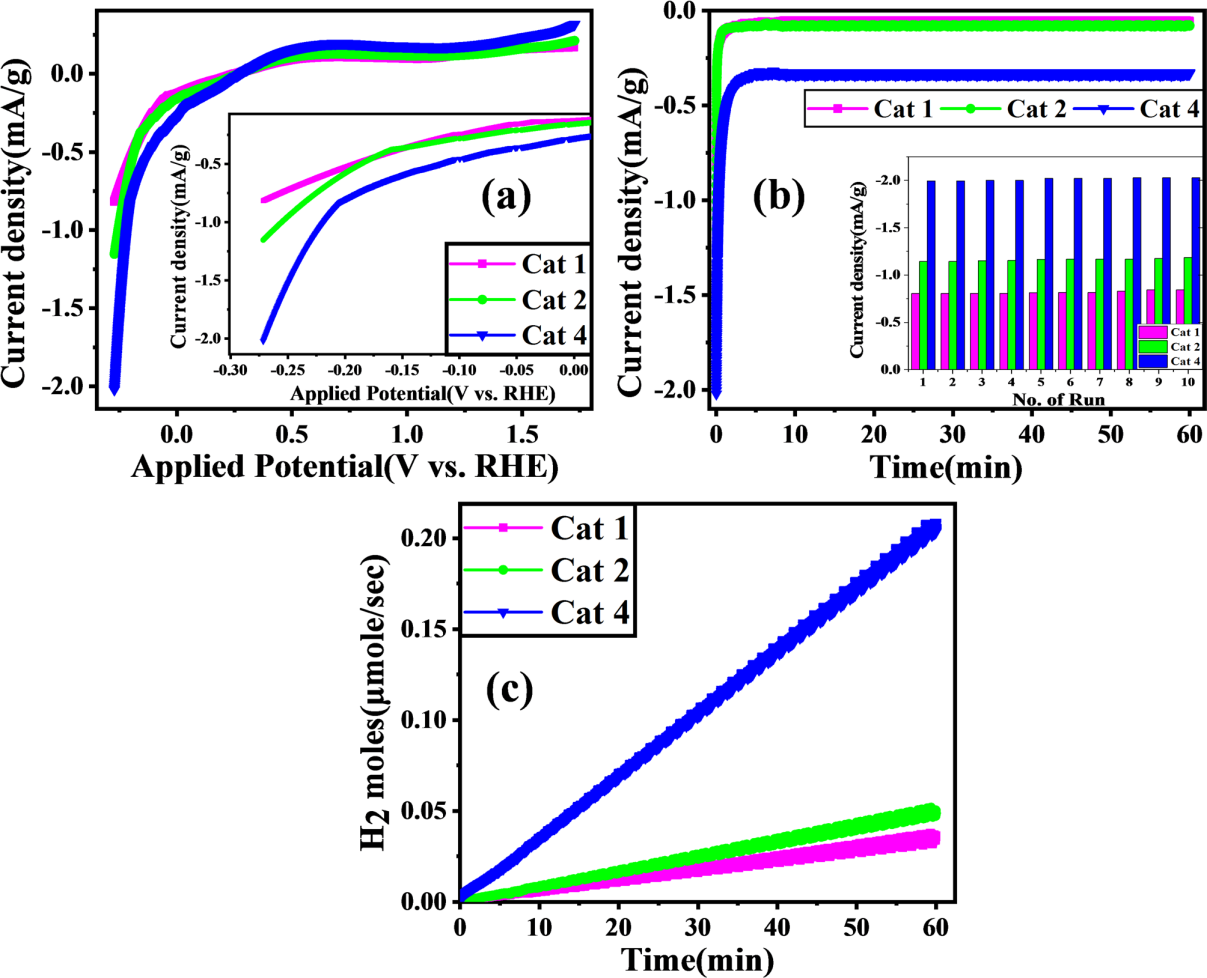
(**a**) Variation of Current density vs. the applied potential vs. RHE, (**b**) Chronoamperometry curves at applied potential vs. RHE, and (**c**) Hydrogen evolution rates.

Figure 5(a) presents the relationship between photocurrent density and applied potential under standard white light illumination (AM 1.5G, 100 mW/cm^2^). The results indicate that all photocatalysts demonstrate a rise in current density as the bias potential becomes more negative. This behavior can be attributed to the improved tunneling of photogenerated charge carriers, facilitating more efficient charge transport^53^. The electrochemical performance of the catalysts, as depicted in the linear sweep voltammetry (LSV) curves, revealed that Cat 4 exhibited a significantly higher photocurrent density compared to Cat 1 and Cat 2. Specifically, Cat 4 achieved a current density of − 2.02 mA/g at − 0.2714 V vs. RHE, demonstrating a substantial improvement over Cat 2 (–1.15 mA/g) and Cat 1 (–0.82 mA/g). This enhanced performance of Cat 4, clearly visualized in the magnified inset of the LSV plot, is attributed to phase transformations that result in an optimized crystal structure, facilitating improved light absorption and efficient charge carrier generation, ultimately leading to superior photocatalytic activity^54,55^.

#### Photocatalyst stability and reusability

The stability and reusability of the photocurrent density were evaluated over 10 consecutive runs, as depicted in the inset of Fig. 5(b). At an applied potential of ‒0.2714 V vs. RHE, Cat 4 exhibited minimal fluctuations, maintaining a current density between ‒1.99 and ‒2.028 mA/g, indicating high stability. Comparable trends were observed for Cat 1 and Cat 2, with slight variations in current density, demonstrating excellent repeatability. Furthermore, the long–term stability of Cat 4 was assessed under continuous illumination for 60 min at ‒0.2714 V vs. RHE (Fig. 5b). An initial decline in current density was observed within the first 1.58 min, reaching approximately ‒1.46 mA/g, after which it stabilized at ‒0.34 mA/g for the remainder of the experiment. The sharp drop in current density for Cat 4 during the first ≈ 2 min in Fig. 5b reflects an initial transient activation/relaxation process, rather than degradation of the material itself. At the start of chronoamperometry, rapid changes in surface charge, double-layer formation, adsorption/desorption of electrolyte species, and the accumulation/detachment of H₂ bubbles can cause a high initial photocurrent that quickly relaxes to a steady-state value. After this early transient, the current density of Cat 4 remains nearly constant (around − 0.3 to − 0.4 mA/g) for the remainder of the 60 min test, and the multicycle J–t runs (inset of Fig. 5b) show highly reproducible behavior over 10 consecutive cycles, indicating that the electrode does not undergo structural or chemical degradation under illumination. Therefore, while we now clarify in the text that there is an initial transient decay in activity, the long-term plateau and reproducible cycling support our conclusion that the material is operationally stable under the tested PEC conditions. These results confirm that the increasing time of ball milling increases the catalyst’s durability and suitability for sustained hydrogen production through the PEC water-splitting^56,57^.

#### Hydrogen evolution rate and solar–to–hydrogen efficiency

The high photogenerated current density under illumination serves as a strong indicator of efficient PEC water splitting, which can be attributed to the band gap energy (Eg) of 2.55 eV in Cat 4. This facilitates a faster redox reaction rate, enhances the tunneling activity of photogenerated carriers, and promotes the PEC process. Theoretically, the hydrogen production from PEC water splitting was estimated using Faraday’s law, as expressed in Eq. (1)^58^

1 $$\mathrm{H}2\left(\mathrm{m}\mathrm{o}\mathrm{l}\mathrm{e}\mathrm{s}\right)=\underset{0}{\overset{\mathrm{t}}{\int}}\frac{{J}_{ph}\mathrm{d}\mathrm{t}}{\mathrm{F}}$$

where F = 9.65$\times$10^4^ C/mol (the Faraday constant) and t is the time of generation.

Based on the recorded photocurrent density over time (Fig. 5b), the corresponding hydrogen evolution data are depicted in Fig. 4c. The results reveal a significant hydrogen generation rate for Cat 1, Cat 2, and Cat 4, with calculated production rates of 0.643, 0.89, and 3.73 mmol/h, respectively. Among them, Cat 4 exhibited the highest hydrogen evolution rate, demonstrating its superior performance in PEC water splitting.

The solar–to–hydrogen (STH) conversion efficiency, a critical metric for evaluating photoelectrochemical water splitting performance, was calculated using Eq. (2)^59^,

2 $$STH=\left[\right(H2/S)\times(237000\left)\right]/\left[100\right]$$

Where H_2_/S is the production rate of the hydrogen moles in mmol/sec. Notably, Cat 4 exhibited a significantly higher STH value of 2.45% compared to Cat 1 (0.42%) and Cat 2 (0.566%). This enhanced STH is attributed to several factors, including improved light absorption efficiency, facilitated by the optimized morphology and band gap energy of Cat 4, which promotes efficient charge carrier generation and transport, leading to increased hydrogen production.

Cat 4 exhibits the highest current density and hydrogen evolution rate because the longer ball-milling duration (6 h) produces a more favorable structural, optical, and electronic configuration for PEC activity. Prolonged milling enhances the dispersion of Co_3_O_4_ within the zeolite framework, increases defect sites, and creates a larger pore volume and wider pore radius, which collectively improve electrolyte accessibility and charge transport pathways. Cat 4 also shows the lowest band gap (2.55 eV), enabling superior visible-light absorption and greater photocarrier generation. The improved Co–O bonding and partial structural reorganization confirmed by FTIR and XRD facilitate faster electron–hole separation and reduce recombination losses. These combined effects allow Cat 4 to utilize incident photons more efficiently, resulting in its markedly higher photocurrent density and hydrogen production rate compared to the other catalysts.

#### Wavelength–dependent photo response and efficiency analysis

**Fig. 6:**
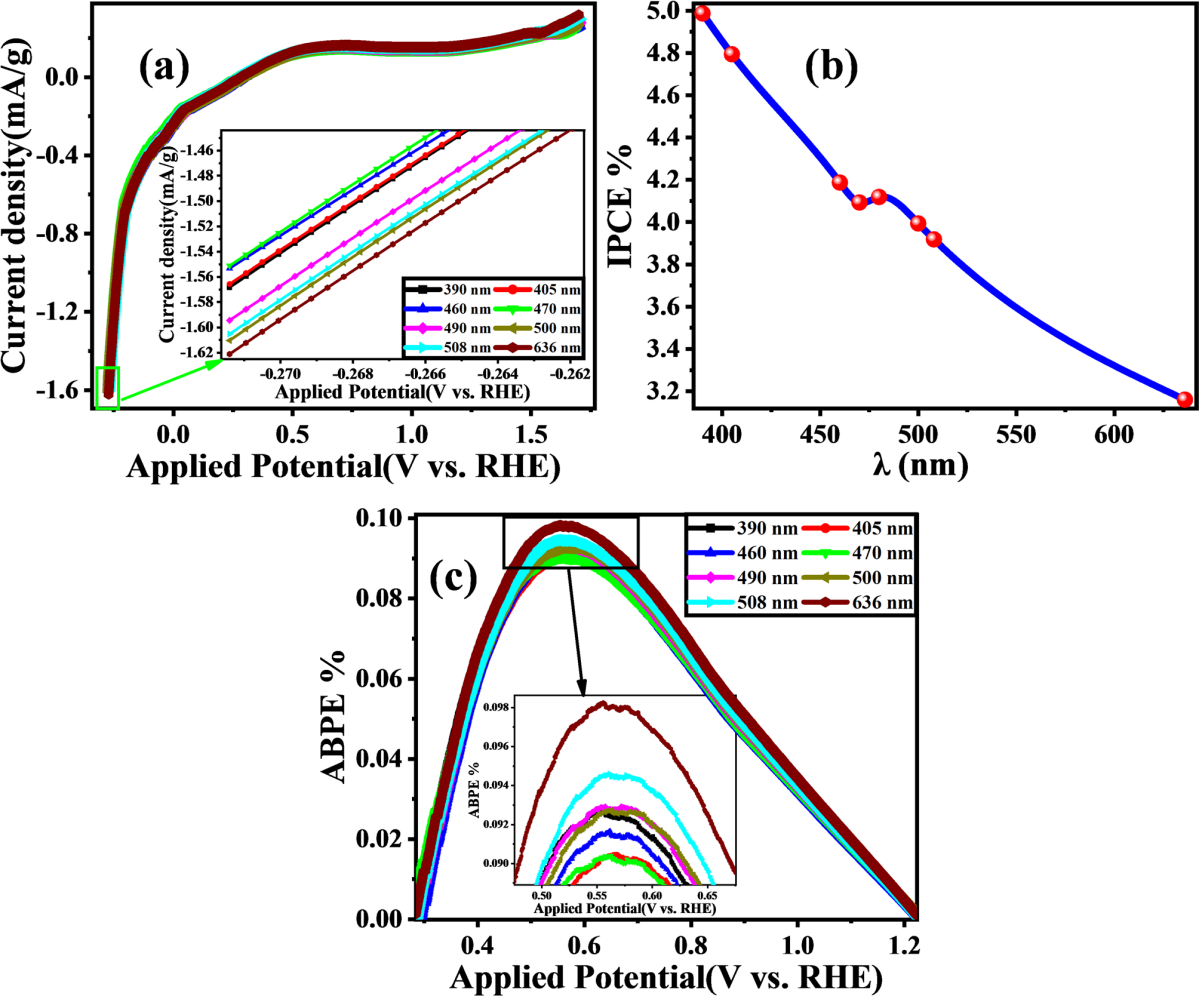
(**a**) Variation of Current density vs. the applied voltage under monochromatic luminance, (**b**) IPCE% %, and (**c**) ABPE% % as a function of applied potential vs. RHE for Cat 4.

To assess the photoelectrochemical response of the Cat 4 photocatalyst under various wavelengths, monochromatic light irradiation ranging from 390 to 636 nm was employed in 0.5 M Na_2_SO_3_·7H_2_O at room temperature, Fig. 6(a). The resulting photocurrent density (Jph) exhibited a wavelength–dependent behavior, reaching a maximum value of − 1.62 mA/g at 636 nm and a minimum of − 1.55 mA/g at 470 nm. This observed trend aligns with the photocatalyst’s light absorption characteristics, confirming its sensitivity to visible light and efficient utilization of solar energy for photoelectrochemical hydrogen production.

The incident photon–to–current conversion efficiency (IPCE%) was determined using Eq. (3)^60^:

3 $$\mathrm{I}\mathrm{P}\mathrm{C}\mathrm{E}\mathrm{\%}=\frac{{\mathrm{J}}_{\mathrm{p}\mathrm{h}}\cdot1240}{{\uplambda}\cdot\mathrm{P}}\times100$$

where J_ph_ represents the photocurrent density, λ is the incident photon wavelength (nm), and P denotes the power density of the monochromatic light (W/m²). As shown in Fig. 6(b), the IPCE% exhibits a wavelength-dependent variation, reaching a maximum of 4.98% at 390 nm and decreasing to 3.16% at 636 nm. The highest value of IPCE% at 390 nm suggests that Cat 4 efficiently absorbs near–UV and visible light, thereby enhancing charge carrier generation and transport, which are crucial for improving its photoelectrochemical performance.

The applied bias photon–to–current efficiency (ABPE%) quantifies the proportion of incident solar energy converted into hydrogen energy, excluding the electrical contribution, and is determined using Eq. (4):

4 $$\mathrm{A}\mathrm{B}\mathrm{P}\mathrm{E}\left(\mathrm{\%}\right)=\frac{{\mathrm{J}}_{\mathrm{p}\mathrm{h}}\cdot({\mathrm{V}}_{\mathrm{a}\mathrm{p}\mathrm{p}}-{\mathrm{V}}_{\mathrm{r}\mathrm{e}\mathrm{v}})}{{\mathrm{P}}_{\mathrm{l}\mathrm{i}\mathrm{g}\mathrm{h}\mathrm{t}}}\times100$$

where V_app_​ is the applied potential (V), V_rev_​ represents the thermodynamic water–splitting potential (1.23 V), and P_light_ denotes the incident light power density (W/m^2^). As illustrated in Fig. 6(c), the highest ABPE of 0.098% was observed at 636 nm with an applied potential of 0.56 V vs. RHE. This performance is attributed to the catalyst’s enhanced charge separation and transport, particularly at shorter wavelengths, where higher photon energy facilitates more efficient photoexcitation, ultimately contributing to improved solar energy conversion into hydrogen^61^.

#### Mott–schottky analysis and PEC/catalytic implications and ICP-OES

**Fig. 7:**
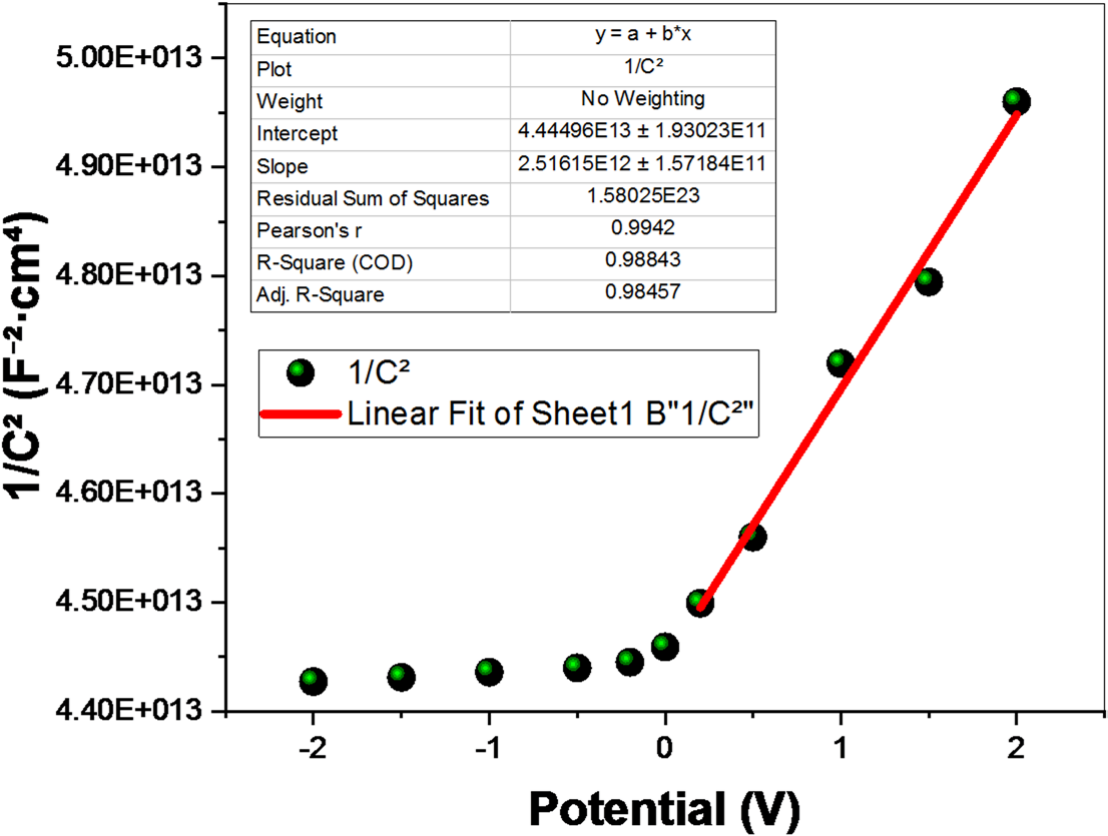
Mott–Schottky plot (1/C² vs. potential) of the Zeo/Co₃O₄ electrode measured at 10 kHz. The plot shows an accumulation region at negative potentials and a linear depletion region between + 0.5 and + 2.0 V vs. Ag/AgCl. Linear fitting of the depletion region yields a flat-band potential of − 1.77 V and an acceptor density of ~ 5 × 10¹⁸ cm⁻³, confirming the p-type semiconducting behavior of the composite.

Electrochemical impedance spectroscopy (EIS) was employed to investigate the semiconductor characteristics of the Zeo/Co₃O₄ electrode through Mott–Schottky (M–S) analysis. Capacitance values were extracted from the high-frequency region (≈ 10 kHz), where the phase angle approached zero and the impedance response was dominated by the space-charge capacitance. These values were normalized to the geometric electrode area (0.5 cm²), and the corresponding (1/C^2^) values were plotted as a function of the applied potential, Fig. 7.

The resulting Mott–Schottky plot exhibited two distinct regions. At negative potentials (–2.0 to 0.0 V vs. Ag/AgCl), the capacitance remained nearly constant, indicating an accumulation regime characteristic of p-type semiconductors. At more positive potential (+ 0.5 to + 2.0 V), a linear region emerged, corresponding to the depletion of majority carriers. This behavior confirms the p-type nature of the Zeo/Co₃O₄ composite, consistent with the well-established electronic structure of cobalt oxide materials, where hole conduction arises from Co²⁺/Co³⁺ redox transitions and oxygen-related defect states^62–64^.

Linear fitting of the depletion region (0.5–2.0 V) using Origin Pro2018 yielded a positive slope of 2.516 × 10¹² F⁻²·cm⁴·V⁻¹ and an intercept of 4.445 × 10¹³ F⁻²·cm⁴ with an excellent correlation coefficient (**R² = 0.988**), further validating the p-type behavior. From the intercept, the flat-band potential (E_fb_) was calculated to be approximately − 1.77 V vs. Ag/AgCl, which aligns well with reported values for Co₃O₄ and related spinel oxides exhibiting deep valence bands and strong oxidative capability^65,66^. Using the slope and the dielectric constant of Co₃O₄ (ε ≈ 12), the acceptor density (N_A_) was estimated to be ~ 5 × 10¹⁸ cm⁻³. This high acceptor density indicates efficient hole transport within the composite and reflects the presence of mixed Co²⁺/Co³⁺ valence states and oxygen-rich defect chemistry that promote p-type conductivity.

These semiconductor properties provide important insight into the catalytic and photoelectrochemical (PEC) behavior of the Zeo/Co₃O₄ electrode. The strongly negative flat-band potential (–1.77 V vs. Ag/AgCl) suggests that the valence band lies at a sufficiently deep energetic position to drive oxidative reactions efficiently, including water oxidation and the transformation of organic or inorganic substrates. Such a negative E_fb_ is consistent with the intrinsic electronic structure of Co₃O₄, where mixed Co²⁺/Co³⁺ valence states and oxygen-rich surface sites contribute to a high density of accessible hole states^62–64^. This energetic alignment facilitates rapid hole accumulation under anodic bias, enabling the electrode to sustain multi-electron oxidation pathways.

The high acceptor density (~ 5 × 10¹⁸ cm⁻³) further supports the strong catalytic activity of the material. A high hole concentration enhances bulk conductivity and reduces charge-transfer resistance at the electrode–electrolyte interface, leading to improved PEC performance. Similar carrier densities have been associated with enhanced oxygen evolution reaction (OER) activity in cobalt-based oxides and spinel structures^65,66^. The observed accumulation behavior at negative potential also suggests that the electrode can efficiently store and mobilize holes, which is advantageous for transient or pulsed PEC processes. Collectively, the Mott–Schottky results demonstrate that the Zeo/Co₃O₄ composite possesses favorable electronic properties—deep valence band, high acceptor density, and efficient hole transport—that contribute to its strong catalytic and PEC performance.

ICP-OES analysis was performed to quantify the cobalt loading in all zeolite/Co₃O₄ composites. For Cat 1–Cat 5, the measured Co concentrations after digestion of 10 mg samples in 100 mL solution were in the range of 21.7–22.3 mg L⁻¹, corresponding to 21.7–22.3 wt% Co in the solids. This translates to a Co₃O₄ content of approximately 29.6–30.4 wt%, in good agreement with the theoretical loading calculated from the synthesis stoichiometry. The close similarity of Co and Co₃O₄ contents across all samples confirms that the ball-milling duration does not significantly affect the cobalt loading, and that differences in PEC performance arise primarily from structural and textural modifications of the zeolite framework rather than variations in Co₃O₄ percentage.

**Table 1:** ICP-OES Measured Co concentration and calculated Co/Co₃O₄ content.

Sample	Co (mg L⁻¹)	Co in solution (mg)	Co wt% in solid	Co₃O₄ wt% in solid*
Cat 1	22.0	2.20	22.0	30.0
Cat 2	21.7	2.17	21.7	29.6
Cat 3	22.3	2.23	22.3	30.4
Cat 4	21.9	2.19	21.9	29.8
Cat 5	22.1	2.21	22.1	30.1

#### Comparison with previously reported co-based catalysts

Tables 1, 2 presents a comparative analysis of various Co_3_O_4_-based catalysts employed in photoelectrochemical (PEC) water splitting, highlighting the unique attributes and effectiveness of the newly studied 6 h-Zeolite/Co_3_O_4_ catalyst (Cat 4)^58–64^. The table includes information on the electrolyte used and current density. Cat 4 stands out due to its notably higher current density and low applied potential, in addition to the simplicity, high throughput, and low cost of the technique used, compared to the other catalysts listed.

**Table 2:** Comparative analysis of Co_3_O_4_-based catalysts and the novel Cat 4 catalyst.

Catalyst	Electrolyte	Current density	Refs.
Co_3_O_4_ nanowires	1 M KOH	−0.50 mA/cm^2^ at − 0.4 V vs. Ag/AgCl	^59^
Co_3_O_4_ nanolayers		−0.45 mA/cm^2^	
Co_3_O_4_/Ag nanowires		−4.73 mA/cm^2^	
Co_3_O_4_/Ag nanolayers		−4.26 mA/cm^2^	
Co_3_O_4_−2/ZnO/FTO nanoplates	0.1 M Na_2_SO_4_ (pH ∼7)	−5.9 mA/cm² at −1.23 V vs. RHE	^60^
BiVO_4_/ZnCo_2_O_4_	0.5 M Na_2_SO_4_	−1.92 mA/cm² at −1.23 V vs. RHE	^61^
Au/Co_3_O_4_/TiO2	0.5 M Na_2_SO_3_	−0.37 mA/cm^2^@1.16 V vs. RHE	^62^
Co_3_O_4_	0.1 M H_2_SO_4_	−1.15 mA/cm^2^ @ −0.4 V vs. RHE	^63^
RGO-Co_3_O_4_	0.1 M H_2_SO_4_	−0.75 mA/cm^2^@−0.35 V vs. RHE	^64^
Ar/Co_3_O_4_/TiO2	0.1 M NaOH	−2.5 mA/cm^2^ @ −1.23 V vs. RHE	^65^
6 h-Zaolite/Co_3_O_4_ (Cat 4)	0.5 M Na_2_SO_3_·H_2_O	–2.02 mA/g at − 0.2714 V vs. RHE	This work

## Conclusion

This study demonstrates the significant influence of ball milling duration on the physicochemical and photoelectrochemical properties of zeolite-Co_3_O_4_ composites. Increased ball milling up to 6 h enhances Co_3_O_4_ dispersion, modifies the zeolite structure, and improves light absorption and charge transport properties. The photoelectrochemical performance of the Cat 4 photocatalyst was systematically evaluated to determine its efficiency in solar-driven hydrogen production. The catalyst exhibited a notable photocurrent response in the visible light spectrum, with the highest photocurrent density observed at 636 nm. The wavelength-dependent IPCE% analysis indicated the material’s strong absorption in the near-UV and visible regions, leading to efficient charge carrier generation. Furthermore, the ABPE% analysis confirmed the catalyst’s capability for effective solar-to-chemical energy conversion, achieving its highest efficiency at 636 nm. Consequently, the catalyst with prolonged ball milling exhibits superior photocatalytic performance for hydrogen evolution. Nevertheless, the solar-to-hydrogen (STH) efficiency achieved (2.45%) remains below the benchmarks required for practical applications. Future optimization of cobalt loading, composite stability, and long-term cycling performance is needed before scale-up. These results underscore the potential of Cat 4 as an efficient photocatalyst for hydrogen evolution applications, paving the way for future studies on optimizing its structural and electronic properties to enhance its overall performance.

## Supplementary Information

Below is the link to the electronic supplementary material.

Supplementary Material 1

## Data Availability

The data presented in this study are available on request from the corresponding author.
