# Supplementary material for: Enhancing photoelectrochemical catalytic performance of zeolite-Co3O4 composites through optimized ball milling duration
Source: Sci Rep. 2026 May 9;16:21483. doi: 10.1038/s41598-026-44358-y (PMC13351014; doi:10.1038/s41598-026-44358-y)
Supplement: Supplementary file 1 — Supplementary Material 1 [file 41598_2026_44358_MOESM1_ESM.docx]

**Supplementary data**

**Enhancing Photoelectrochemical Catalytic Performance of Zeolite-Co_3_O_4_ Composites through Optimized Ball Milling Duration**

**Ghadah M. Al-Senani^1^, Mohamed Shaban^2^***,  **Salhah D. Al-Qahtani^1^, Khaled Abdelkarem^3^**^‡^**, Rana Saad^4^**^‡,^***,**

^1^Department of Chemistry, College of Science, Princess Nourah bint Abdulrahman University, P.O. Box 84428, Riyadh 11671, Saudi Arabia; [gmalsnany@pnu.edu.sa](mailto:gmalsnany@pnu.edu.sa) (G.M.A.); [sdalohtany@pnu.edu.sa](mailto:sdalohtany@pnu.edu.sa) (S.D.A.)

^2^Department of Physics, Faculty of Science, Islamic University of Madinah, P. O. Box: 170, Madinah 42351, Saudi Arabia; mssfadel@iu.edu.sa (M.S.)

^3^Department of Physics, Chonnam National University, Gwangju 61186, Republic of Korea. E-mail: [oldfighter.khaled123@gmail.com (K.A.)](mailto:oldfighter.khaled123@gmail.com(K.A.))

^4^Nanophotonics and Applications (NPA) Lab, Department of Physics, Faculty of Science, Beni-Suef University, Beni-Suef 62514, Egypt; [ranasaad811@gmail.com (R.S.)](mailto:ranasaad811@gmail.com(R.S.))

^‡^ Both authors equally contributed to this work

**^*^Correspondence:** [**mssfadel@iu.edu.sa**](mailto:mssfadel@iu.edu.sa) **(M.S.)** [ranasaad811@gmail.com (R.S.)](mailto:ranasaad811@gmail.com(R.S.))

**Table 1:** Surface Area, Pore Volume, and Pore Radius of Different Catalysts.

| Catalyst | BET surface area (m²/g) | BJH | DFT method | Total Pore Volume at relative pressure 0.99540 | Average Pore radius (nm) |
| --- | --- | --- | --- | --- | --- |
| Cat 1 | 72.21 | Pore Volume 0.126833 cc/g  Surface Area 43.5787 m²/g  Pore radius Dv(r) 1.92384 nm | Pore Volume 0.1305 cc/g  Surface Area 51.0182 m²/g | 0.13881cc/g for pores < 209.24 nm | 3.8447 nm |
| Cat 2 | 108.751 | Pore Volume 0.173908 cc/g Surface Area 59.416 m²/g  Pore radius Dv(r) 1.92383 nm | Pore Volume 0.1798 cc/g  Surface Area 69.0223 m²/g | 0.18991 cc/g for pores < 131.55 nm | 3.4927 nm |
| Cat 3 | 131.665 | Pore Volume 0.132971 cc/g Surface Area 62.5296 m²/g  Pore radius Dv(r) 1.91936 nm | Pore Volume 0.1418 cc/g  Surface Area 68.7803 m²/g | 0.14958 cc/g for pores < 201.90 nm | 2.2722 nm |
| Cat 4 | 47.0813 | Pore Volume 0.231112 cc/g Surface Area 38.4991 m²/g  Pore radius Dv(r) 1.92884 nm | Pore Volume 0.2259 cc/g  Surface Area 52.6778 m²/g | 0.23884 cc/g for pores < 105.32 nm | 10.146 nm |
| Cat 5 | 54.0373 | Pore Volume 0.153967 cc/g Surface Area 43.9978 m²/g  Pore radius Dv(r) 1.93569 nm | Pore Volume 0.1527 cc/g  Surface Area 50.8053 m²/g | 0.16242 cc/g for pores < 165.82 nm | 6.0115 nm |

**Figure S1.** The current density vs. the applied potential of the different samples. The inset shows the current density at -0.2714 V vs. RHE.

**
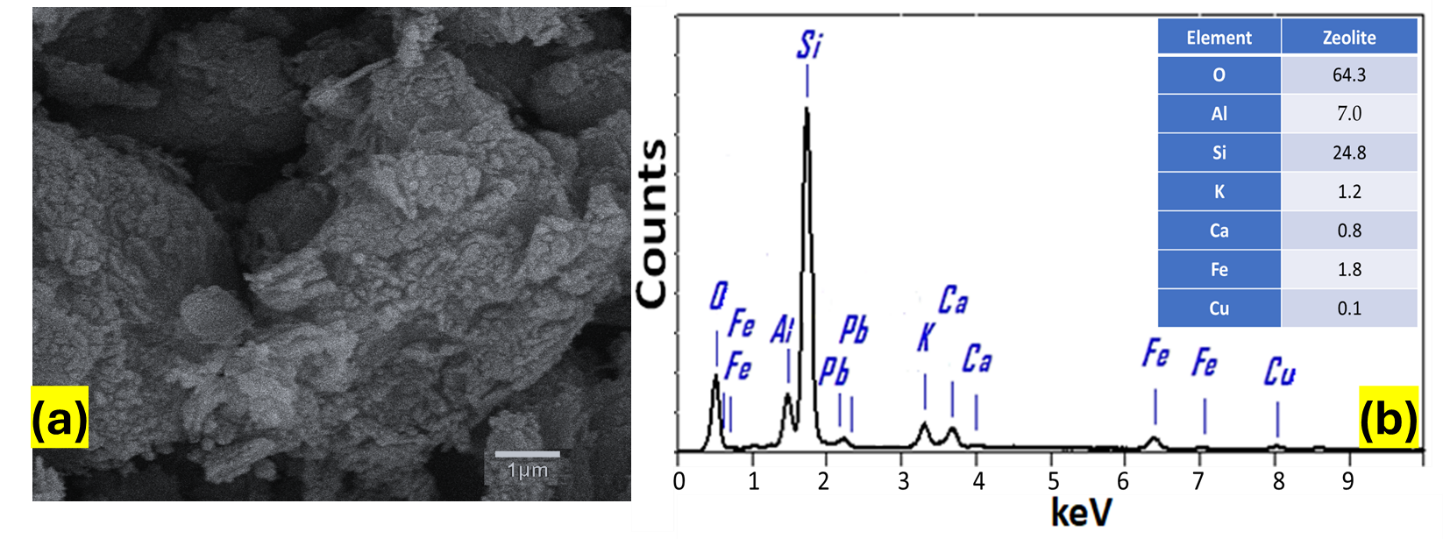
**

**Figure S2.** (a) SEM image and (b) EDX chart of natural zeolite.

**Figure S3.** XRD chart of Cat 3.
